# The role of IP_3_R and TRPC3 channels in vasoconstriction and hypertension

**DOI:** 10.1007/s00424-026-03175-y

**Published:** 2026-05-02

**Authors:** Raiana dos Anjos Moraes, Quiara Lovatti Alves, Liliane Barreto da Silva, Rafael Leonne Cruz de Jesus, Darízy Flávia Silva

**Affiliations:** 1 Postgraduate Program in Biotechnology in Health and Investigative Medicine (PgBSMI), Gonçalo Moniz Institute, FIOCRUZ, Salvador, BA Brazil; 2 Laboratory of Cardiovascular Physiology and Pharmacology, Bioregulation Department, Federal University of Bahia, Salvador, BA Brazil; 3 Cardiovascular Translational Research Center, School of Medicine, University of South Carolina, Columbia, SC USA; 4 Biomedical Engineering Program, University of South Carolina, Columbia, SC USA

**Keywords:** Hypertension, Inositol 1,4,5-trisphosphate, Inositol 1,4,5-trisphosphate receptor, Transient receptor potential canonical type 3 channel, Vasoconstriction

## Abstract

Hypertension is a major global health concern and a leading risk factor for cardiovascular diseases, including stroke, myocardial infarction, and heart failure. A hallmark of hypertension is elevated total peripheral vascular resistance, often driven by sustained and abnormal vasoconstriction. Calcium ions (Ca²⁺) play a central role in vascular smooth muscle cell (VSMC) contraction, and their intracellular concentration is tightly regulated by multiple signaling pathways. Among these, the inositol 1,4,5-trisphosphate receptor (IP_3_R) and the transient receptor potential canonical type 3 (TRPC3) channel are critical mediators of Ca²⁺ signaling. IP_3_R activation triggers Ca²⁺ release from the endoplasmic reticulum, while TRPC3 channels facilitate Ca²⁺ and Na⁺ influx across the plasma membrane. Several studies have shown that both IP_3_Rs and TRPC3 channels are upregulated in hypertensive animal models. Human studies have also demonstrated elevated TRPC3 expression in the context of pulmonary arterial hypertension (PAH). This review provides a comprehensive overview of the structural domains and membrane microdomains that facilitate IP_3_R–TRPC3 coupling and Ca²⁺ influx. IP₃ and endothelin-1 stimulate TRPC3 channels and promote their molecular coupling to IP_3_Rs, leading to activation of nonselective cation currents in artery myocytes. Increased expression and/or activity of IP_3_Rs and TRPC3 channels amplifies this signaling, contributing to the increased vascular tone characteristic of the hypertensive state. Understanding the molecular interplay between IP_3_Rs and TRPC3 channels offers new insight into the dysregulated Ca²⁺ signaling underlying hypertension. Targeting this coupling mechanism may represent a novel therapeutic strategy to restore vascular homeostasis and reduce blood pressure in affected individuals.

## Introduction

Arterial blood pressure is a highly regulated physiological parameter that relies on multiple mechanisms to maintain adequate blood flow, ensuring the delivery of oxygen and nutrients to all organs and tissues according to their metabolic demands. Three main key physiological mechanisms are involved in blood pressure regulation: the contraction of small arteries, renal control of blood volume, and the modulation of cardiac output [1]. This regulation is multifactorial and involves cardiovascular, neural, endocrine, and genetic components, among others [2]. Disruption of these regulatory mechanisms can result in sustained elevations in arterial pressure, ultimately leading to end-organ damage [3]. Hypertension is a chronic medical condition widely recognized as the leading modifiable risk factor for cardiovascular disease [2] and remains a major contributor to cardiovascular morbidity and mortality worldwide [4]. Over the past three decades, the global prevalence of hypertension has risen markedly, with the number of affected adults increasing from approximately 650 million in 1990 to 1.3 billion in 2019 [5]. Given this alarming trend, understanding the underlying physiological mechanisms is essential. Blood pressure is determined primarily by cardiac output and total peripheral resistance [6], with resistance arteries and downstream arterioles playing a central role in the regulation of vascular resistance and blood pressure [7]. Increased peripheral resistance is primarily driven by enhanced vasoconstriction and dysregulated vascular signaling pathways, which represent key molecular mechanisms underlying vascular dysfunction and the development of hypertension [8]. Ion channels, such as calcium channels, play a fundamental role in this process, as intracellular calcium concentration directly influences the contractile activity of vascular smooth muscle cells (VSMC) [9]. Among these, the inositol 1,4,5-trisphosphate receptor (IP_3_R), predominantly localized in the membrane of the endoplasmic reticulum (ER), is particularly important in regulating VSMC contraction. However, the precise role of intracellular Ca²⁺ release channels, such as IP_3_R, in blood pressure homeostasis remains incompletely understood [10]. Additionally, transient receptor potential canonical type 3 (TRPC3) channels have been identified as significant contributors to the pathogenesis of hypertension [11, 12]. This review focuses on the concept that TRPC3 channels and IP₃ receptors do not function as independent Ca²⁺ signaling pathways, but rather form a functionally coupled microdomain signaling unit within ER–plasma membrane junctions. We discuss how this interaction integrates intracellular Ca²⁺ release with membrane depolarization and Ca²⁺ influx, thereby amplifying vasoconstrictor responses. In addition, we highlight how dysregulation of this coupling may contribute to vascular dysfunction in hypertension and represent a potential target for more selective therapeutic strategies.

### Ca²⁺ signaling in vascular dysfunction and hypertension

Ca²⁺ signaling represents a key regulatory mechanism controlling endothelial function, vascular smooth muscle contractility, and the cellular and extracellular processes involved in vascular remodeling. The cytosolic free Ca²⁺ concentration is precisely maintained through the coordinated regulation of Ca²⁺ mobilization and removal processes. Calcium can be released from intracellular stores within the sarcoplasmic and endoplasmic reticulum or enter the cytosol through voltage-gated, transient receptor potential (TRP), and store-operated Ca²⁺ channels. Restoration of basal Ca²⁺ levels is achieved by active extrusion through the plasma membrane Ca²⁺-ATPase (PMCA) and the Na⁺/Ca²⁺ exchanger (NCX), as well as by reuptake into the sarco(endo)plasmic reticulum via the Ca²⁺-ATPase (SERCA) and mitochondrial Ca²⁺ uptake systems [13].

Acute increases in intracellular Ca²⁺ are triggered by stimuli such as angiotensin II [14], norepinephrine, and 5-hydroxytryptamine [15]. However, sustained exposure to pro-hypertensive stress (chronic neurohumoral stimulation, increased blood flow, and/or pressure overload) induces gene-expression changes in both VSMCs and endothelial cells, leading to vascular remodeling. This process involves coordinated structural and functional changes [16, 17]. Functional remodeling is characterized by changes in ion channel expression and activity and reorganization of Ca²⁺ microdomain components in VSMCs, which can amplify vasoconstrictor signaling [18]. In clinical conditions such as hypertension, vascular inflammation, atherosclerosis, and coronary artery disease, intracellular Ca²⁺ homeostasis in VSMCs is altered [13]. Dysregulation of intracellular Ca²⁺ arises from multiple convergent mechanisms. One such mechanism involves the upregulation of TRPC3, which alters the composition of TRPC3/TRPC6 heteromultimers, increasing the proportion of TRPC3 subunits in hypertensive vascular smooth muscle cells. Because TRPC3 channels exhibit weak inward rectification, this shift enhances basal current at negative potential, leading to membrane depolarization, elevated intracellular Ca²⁺, and increased basal tone [19].

In parallel, stromal interaction molecules (STIMs) and Orai proteins mediate store-operated Ca²⁺ entry (SOCE), another major pathway of Ca²⁺ influx. Expression of STIM1 and Orai1 is upregulated in the aorta of hypertensive rats, indicating that enhanced STIM1/Orai1-dependent SOCE contributes to Ca²⁺ overload and abnormal vasoconstrictor responses in hypertension [20].

Additionally, NADPH oxidase 5 (Nox5) dysregulation can induce oxidative stress and augment Ca²⁺ signaling, leading to increased vasoconstriction and vascular dysfunction [21]. Furthermore, oxidative stress activates redox-sensitive TRPM2 channels, which enhance Ca²⁺ and Na⁺ influx and promote reverse-mode NCX activation, amplifying intracellular Ca²⁺ accumulation and vascular contractility [22].

Another important mechanism involves increased production of inositol 1,4,5-trisphosphate (IP₃), which activates IP_3_Rs [23, 24] and/or increases TRPC3 channel expression [12, 25]. Recent evidence suggests that this amplification may depend not only on individual signaling pathways, but also on functional coupling between IP_3_Rs and TRPC3 channels within specialized microdomains.

### IP₃ Receptors: structure and regulation

IP_3_ and diacylglycerol (DAG) are secondary messengers formed by the cleavage of phosphatidylinositol 4,5-bisphosphate (PIP_2_), a component of the plasma membrane, through phospholipase C [26, 27]. In 1983, a study demonstrated that IP_3_ mobilizes Ca^2+^ from nonmitochondrial intracellular Ca^2+^ stores in pancreatic acinar cells [28]. Subsequently, other work observed that IP₃ induces the opening of Ca²⁺ channels in lipid bilayers incorporated with vesicles formed from the sarcoplasmic reticulum of vascular smooth muscle [29]. Thereafter, the IP_3_R was isolated and purified from bovine aorta [30]. Structurally, each IP_3_R1 subunit comprises ten distinct domains that are organized within the tetrameric channel around a central fourfold axis, contributing to channel stability, ion conduction, ligand binding, and regulatory functions. Approximately 90% of the protein extends into the cytosol, including most of the N-terminal region and the C-terminal tail, while the remaining portion forms the transmembrane and luminal domains. The ion-conducting gate is located at the point of constriction along the TM6 helices [31].

Three isoforms of the IP_3_R have been identified, which assemble into homo- and heterotetrameric channels. These isoforms are often co-expressed within the same cells, and their tissue-specific distribution reflects their diverse biological functions [32, 33]. The IP_3_R type I isoform is predominantly expressed in the central nervous system, kidney [34], cerebral artery smooth muscle cells [35], aorta, as well as basilar and mesenteric arteries of the rat [36]. In contrast, IP_3_R type II shows high expression levels in the heart, skeletal muscle, liver, and kidney, while IP_3_R type III is mainly found in the testis, thymus, and pancreas 34.

In addition, IP_3_Rs interact with numerous regulatory proteins, including Bcl-2 (B-cell lymphoma 2), CaM (calmodulin), IRBIT (IP₃-binding protein released with IP₃), and TRPC channels, which associate with cytosolic or luminal domains of the receptor to regulate its distribution, coupling to signaling pathways, sensitivity to IP₃ and Ca²⁺, and the targeted delivery of Ca²⁺ to specific effector proteins [32].

IP_3_Rs activity is modulated by several factors, including Ca^2+^, ATP [37], and phosphorylation [38]. Cryo-EM structures of human IP_3_R3 reveal multiple gating conformations from a single dataset, including IP₃–ATP-bound pre-active closed states, an IP₃–ATP–Ca²⁺-bound open state, and an IP₃–ATP–Ca²⁺-bound inactive closed state. These results demonstrate that channel gating is governed by the allosteric interplay of IP₃, Ca²⁺, and ATP, with IP_3_Rs coactivated by IP₃ and Ca²⁺, inhibited by high Ca²⁺, and potentiated by ATP [39]. At elevated concentrations, Ca²⁺ inhibits IP_3_R activity, thereby establishing a negative feedback mechanism that contributes to the precise spatiotemporal regulation of intracellular Ca²⁺ signals [40]. Beyond regulation by Ca²⁺, ATP, and phosphorylation, evidence over the past few years has identified miR-204 as a regulator of IP_3_R1 in vascular smooth muscle. In mice lacking miR-204, aortas and mesenteric resistance arteries exhibit heightened vasoconstrictor responses to multiple agonists and an exaggerated pressor response to angiotensin II, despite similar basal systolic blood pressure. These functional changes are accompanied by increased IP_3_R1 expression in the vasculature, and the enhanced agonist-induced vasoconstriction is abolished by pharmacological inhibition of IP_3_R1 signaling. In vitro, miR-204 inhibition increases IP_3_R1 and enhances agonist-evoked SR Ca²⁺ release, whereas miR-204 overexpression downregulates IP_3_R1 [41].

Recent work in intact resistance arteries indicates that mitochondria modulate IP_3_R-dependent Ca²⁺ release recruited during voltage-dependent Ca²⁺ entry. Specifically, Ca²⁺ entry through voltage-dependent channels generates two distinct cytosolic Ca²⁺ components: a slow, sustained increase attributable to Ca²⁺ influx and rapid repetitive Ca²⁺ oscillations and propagating waves that require Ca²⁺ release from internal stores via IP_3_Rs. Notably, mitochondria exert little influence on the slow component but strongly regulate the IP_3_R-dependent oscillatory/wave component of the signal [42].

### TRPC3 Channels: structure and regulation

The TRP superfamily comprises a diverse group of non-selective cation channels broadly conserved across species. In mammals, TRP channels exhibit considerable diversity in their activation mechanisms, ion permeability, and physiological roles [43]. TRPC3 channels exhibit the conserved structural features of TRP channels, characterized by N-terminal ankyrin repeat domains, six transmembrane segments with a pore-forming loop between S5 and S6, and a C-terminal EWKFAR motif, referred to as the TRP box [44]. The cytoplasmic domain of TRPC3 functions as a regulatory sensor that interprets intracellular signals to modulate channel activity. Notably, its C-terminal region contains a CaM/IP₃ receptor–binding (CIRB) site. Binding of Ca²⁺–CaM to the CIRB inhibits TRPC3 activity, whereas under low Ca²⁺ conditions, the IP₃ receptor competes for this site, promoting channel activation [45]. These channels exhibit relatively low selectivity for Ca²⁺ over Na⁺ [46].

Cryo-EM analyses of human TRPC3 show that the TRP helix is disengaged from the pore-lining S6; instead, it is positioned near the S4–S5 linker. The unusually extended S3 helix shapes an atypical transmembrane architecture and contributes to an extracellular region that contains a cavity with the potential to accommodate small molecules, consistent with a possible role in sensing extracellular stimuli. These structures also reveal two lipid-interaction sites: one located in a pocket formed by the pre-S1 “elbow,” S1, and the S4–S5 linker, and a second positioned near the pore domain, supporting a potential link between lipid binding and channel activation [47].

In addition, three putative Ca²⁺-binding sites (CBS1–CBS3) have been described for TRPC3. Experimental evidence indicates that antagonistic effects of CBS1 (inhibitory) and CBS3 (activating) contribute to controlling channel activity [48, 49]. TRPC3/6/7 subunits can form homo- or heterotetrameric channels, which can modify channel gating and ion permeation properties [50].

TRPC3 channels can be activated by stimulation of phospholipase C-coupled receptors [44, 51, 52]. Phosphatidylinositol 4,5-bisphosphate [PI(4,5)P₂] can also regulate TRPC3 function. First, PI(4,5)P₂ is required for recruitment and retention of TRPC3 within endoplasmic reticulum–plasma membrane (ER-PM) junctions, involving an N-terminal two phenylalanines in an acidic tract (FFAT) motif at the TRPC3 N-terminal loop within the linker helices that envelope the C-terminus pole helix. Second, PLC-dependent PI(4,5)P₂ hydrolysis generates DAG, which engages the pore-associated lipid site (site 2) and is important for receptor-mediated channel activation, which is influenced by ER-PM junction localization. Third, PI(4,5)P₂ binding at site 1 modulates TRPC3 activity and controls the interaction of the lipid with site 2. STIM1 is important for ER-PM junctions, enhancing ER-PM communication and mutual regulation [53].

TRPC3 channels also participate in functional signaling complexes through dynamic or stable interactions with other ion channels and regulatory proteins. These include coupling to IP_3_Rs and physical interactions with NCX proteins [48, 54, 55]. The formation of such complexes contributes to the precise regulation of TRPC3 activity, allowing for context-dependent regulation based on expression levels, cell type, and signaling environment [56, 57].

### TRPC3–IP_3_R Coupling: microdomain organization

Previous studies have demonstrated a functional interaction between IP_3_Rs and store-operated TRPC3 channels. Kiselyov et al. first noted that cytoplasmic IP₃-binding domains could productively interact with TRPC3 in HEK293 cells [58, 59. Further characterization of this interaction, using coimmunoprecipitation and glutathione S-transferase (GST) pulldown techniques, identified two TRP-interacting sequences (F2q and F2g) in IP_3_R that associate with a region of TRP, C7 [60]. In parallel, Birnbaumer and colleagues discussed evidence suggesting interactions between IP_3_Rs and TRPC3 [61]. Interestingly, IP_3_Rs and Ca²⁺/CaM appear to compete for binding to the CIRB domain of TRPC3, suggesting that Ca²⁺/CaM exerts an inhibitory effect on TRPC3 function [62, 63]. However, work by Wedel et al. demonstrated that, although the CIRB domain influences plasma membrane targeting of TRPC3, this process is independent of direct IP_3_R binding. Moreover, TRPC3-mediated cation entry persists in cells lacking all IP_3_R isoforms, indicating that IP_3_Rs are not strictly required for TRPC3 activation [64]. Efforts to identify the structural components facilitating local communication between IP_3_Rs and TRPC3 have highlighted the role of membrane microdomains. Lockwich et al. reported that TRPC3 assembles within caveolar Ca²⁺ signaling complexes, and that caveolar invaginations may facilitate close proximity between TRPC3 and IP_3_R1. Additionally, remodeling of the actin cytoskeleton influences the localization of the TRPC3-associated signaling complex, highlighting the role of cytoskeletal dynamics in organizing these signaling domains [65]. Moreover, caveolin-1 (Cav-1) has been shown to colocalize sarcoplasmic reticulum IP_3_R1 and plasma membrane TRPC3 channels in smooth muscle cells from resistance-sized cerebral arteries. The Cav-1 scaffolding domain (CSD) can interact with CSD-interacting motifs located on both the N- and C-terminal regions of IP_3_R1, as well as the C-terminus of TRPC3 [66].

In addition to caveolar scaffolding, Treves and colleagues provided evidence that junctate–IP_3_R–TRPC3 supramolecular complexes contribute to stable ER–PM peripheral junctions. These structures may serve as anatomical sites for agonist-activated Ca²⁺ entry [67]. To the best of our knowledge, the first demonstration of molecular coupling between IP_3_R and TRPC3 in native cells was provided by Adebiyi and colleagues. In cerebral artery myocytes, IP_3_R1 resides in close spatial proximity to TRPC3 channels, as demonstrated by immunofluorescence resonance energy transfer (immuno-FRET) microscopy, suggesting selective coupling between these proteins. IP_3_ binding enhances the physical interaction between the N-terminus of IP_3_R1 and the CIRB domain of TRPC3, promoting plasma membrane cation influx and contributing to IP_3_-induced vasoconstriction [68]. More recently, Curcic et al. reported that TIRF imaging demonstrated dynamic colocalization between TRPC3 and IP_3_R at ER–PM junctions, supporting their interaction within these microdomains [69].

Together, these findings support a model in which TRPC3–IP_3_R coupling operates as a dynamic signaling complex within ER–PM microdomains, characterized by precise spatial organization and dynamic protein interactions (Table 1).

**Table 1:** Key studies demonstrating TRPC3–IP_3_R interaction

Authors	Model/System	Key Finding
Kiselyov et al., (1998/1999) [58, 59]	HEK293 cells	First evidence of functional interaction between IP_3_R and TRPC3
Boulay et al., (1999) [60]	HEK293 cells	TRP-interacting sequences (F2q and F2g) in IP_3_R that associate with a region of TRP, C7.
Zhang et al., (2001) [63]	HEK293 cells	IP_3_R competes with Ca²⁺/CaM for binding to TRPC3 (CIRB domain)
Lockwich et al., 2001 [65]	HEK293 cells	TRPC3 is assembled in caveolar Ca²⁺ signaling complexes associated with IP_3_R
Zhu & Tang, (2004) [62]	HEK293 cells	IP_3_R and calmodulin compete for binding to TRPC3, regulating channel activity
Treves et al., 2010 [67]	HEK293 cells	Junctate–IP_3_R–TRPC3 complexes form stable ER–PM junctions
Adebiyi et al., 2010 [68]	Cerebral artery myocytes	First demonstration of TRPC3–IP_3_R coupling in native vascular smooth muscle cells. Close spatial proximity between IP_3_R and TRPC3 establishes this isoform-selective functional interaction.
Adebiyi et al., 2011 [66]	Smooth muscle cells of resistance-size cerebral arteries	Caveolin-1 assembles IP_3_R and TRPC3 into a functional signaling complex in arterial smooth muscle cells
Curcic et al., 2022 [69]	HEK293 cells	Dynamic TRPC3–IP_3_R colocalization at ER–PM junctions

### Functional impact of TRPC3–IP_3_R Coupling in vascular smooth muscle

**Fig. 1:**
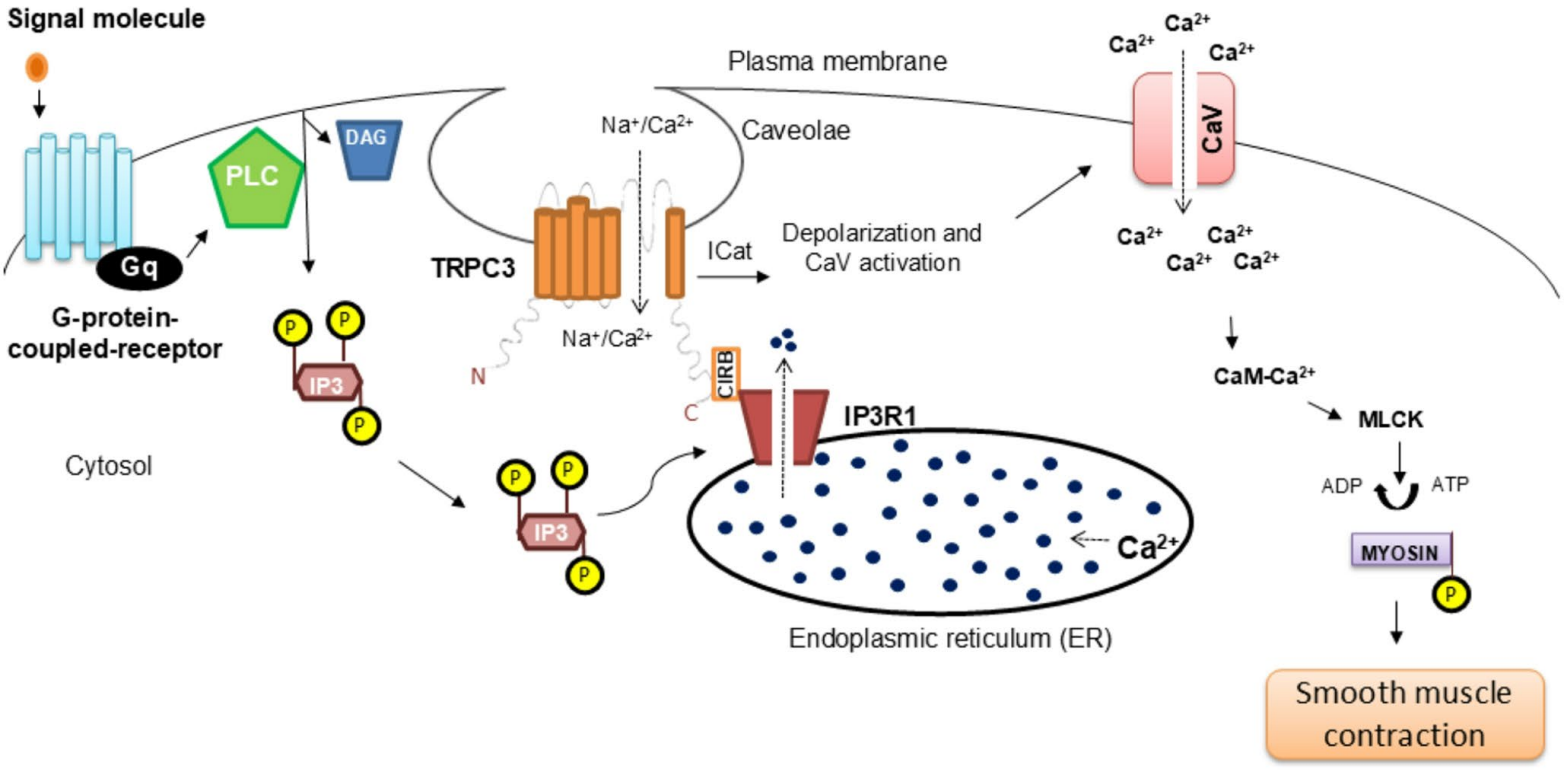
Proposed model of TRPC3–IP_3_R microdomain coupling in vascular smooth muscle. Agonist binding to G protein–coupled receptors activates phospholipase C (PLC), leading to cleavage of phosphatidylinositol 4,5-bisphosphate (PIP_3_) and generation of inositol 1,4,5-trisphosphate (IP₃) and diacylglycerol (DAG). IP₃ binds to IP₃ receptors on the endoplasmic reticulum (ER), promoting Ca²⁺ release into the cytosol. In addition, IP₃ can elicit a nonselective cation current (ICat) through functional coupling between IP_3_R1 and TRPC3, resulting predominantly in Na⁺ influx, membrane depolarization, and activation of voltage-dependent Ca²⁺ channels (CaV), thereby further elevating intracellular Ca²⁺. Increased cytosolic Ca²⁺ binds calmodulin (CaM) to activate myosin light chain kinase (MLCK), which phosphorylates myosin and promotes smooth muscle contraction. The hypertensive panel illustrates enhanced TRPC3/IP_3_R1-driven Ca²⁺ signaling and increased smooth muscle contraction, as indicated by the red arrows. TRPC3, transient receptor potential canonical 3; CaV, voltage-dependent Ca²⁺ channels; ER, endoplasmic reticulum; MLCK, myosin light chain kinase; IP₃, inositol 1,4,5-trisphosphate; IP_3_R1, inositol 1,4,5-trisphosphate receptor type 1; CIRB, calmodulin/IP_3_ receptor-binding site; PLC, phospholipase C; CaM, calmodulin; ICat, cation current; DAG, diacylglycerol; Na⁺, sodium; Ca²⁺, calcium; P, phosphate

The spatial organization of TRPC3–IP_3_R coupling provides the structural basis for its functional impact in vascular smooth muscle. Functionally, this coupling links intracellular Ca²⁺ release to plasma membrane ion influx. A compelling mechanism by which IP₃ modulates vascular tone involves IP_3_R-mediated activation of TRPC3 channels. Transient or sustained elevations in intracellular IP_3_ levels stimulate IP_3_R-mediated TRPC3 channel activation, resulting in nonselective cation currents, primarily due to Na⁺ influx. This leads to membrane depolarization, activation of voltage-gated Ca²⁺ channels, increased intracellular calcium concentrations, and ultimately vasoconstriction [70] (Fig. 1).

In a related context, Song et al. provided evidence that IP_3_ can directly activate nonselective cation channels in airway smooth muscle cells. Their findings support a model in which IP₃ binding may directly activate TRPC3 channels, promoting extracellular Ca²⁺ entry [71].

Curcic et al. reported that in HEK293T cells, calcium influx via TRPC3 channels, localized at ER–PM junctions, modulates IP_3_R activity. TRPC3-mediated Ca²⁺ entry regulates IP_3_R function at these sites, shaping Ca²⁺ signaling dynamics and facilitating selective activation of downstream effectors. Notably, when TRPC3-driven local Ca²⁺ influx exceeds a critical threshold, it suppresses IP_3_R-dependent Ca²⁺ oscillations, indicating dynamic, Ca²⁺-dependent feedback between TRPC3 and IP_3_Rs [69]. Although these findings were obtained in non-vascular cells, they provide important mechanistic insights that may be relevant to vascular smooth muscle physiology. It will be important for future studies to determine whether similar TRPC3–IP_3_R regulatory mechanisms operate in vascular smooth muscle cells under physiological and pathological conditions.

The efficiency of this coupling depends on the integrity of the underlying microdomain architecture. Structures such as ER–PM junctions, caveolar complexes, and cytoskeletal elements facilitate close proximity between IP_3_Rs and TRPC3 channels, enabling rapid and coordinated signaling [53, 65–67. Disruption or remodeling of these structures may therefore significantly alter the strength and duration of TRPC3–I_3_R communication. In pathological conditions such as hypertension, increased expression and/or activity of TRPC3 channels and IP_3_Rs has been associated with enhanced coupling efficiency and augmented vasoconstrictor responses [25]. Together, these findings support the concept that TRPC3–IP_3_R coupling functions as a localized signaling module that amplifies vasoconstrictor signaling in vascular smooth muscle cells.

### Enhanced IP_3_R and TRPC3 signaling in hypertensive vasculature

Hypertension is partly driven by increased peripheral vascular resistance, which results from abnormal vascular contraction. This dysfunction is closely associated with altered calcium signaling, including enhanced calcium influx, increased calcium release from the sarcoplasmic reticulum, upregulation of calcium influx channels, and heightened responsiveness to vasoconstrictor agonists under hypertensive conditions [8, 72, 73]. Pioneering research by Liu and colleagues demonstrated increased TRPC3 channel expression and elevated TRPC3-mediated calcium influx in monocytes from spontaneously hypertensive rats (SHR) [74] and essential hypertensive patients [75]. Subsequently, they provided the first evidence of enhanced TRPC3 protein expression in the aorta of adult SHR compared with Wistar Kyoto (WKY) controls. This upregulation correlated with increased vasoconstriction, calcium influx, and blood pressure elevation induced by angiotensin II in vascular smooth muscle cells (VSMCs) from SHR [12]. Supporting these findings, increased TRPC3 expression was also observed in mesenteric arterioles from SHR, promoting enhanced oscillations in vascular tone associated with hypertension [76]. Similarly, TRPC3 protein levels are elevated in the carotid artery of SHR, resulting in augmented Ca²⁺ and Na⁺ influx [77]. Álvarez-Miguel et al. reported a greater contribution of TRPC3 channels, both as homo- and heterotetramers with TRPC6, in VSMCs from blood pressure-high (BPH) mice, potentially facilitating cell depolarization [19]. Beyond plasma membrane Ca²⁺ entry, TRPC3 has also been implicated in mitochondrial Ca²⁺ handling in hypertensive vessels. In SHR, TRPC3 expression was elevated in purified vascular mitochondrial fractions and was associated with increased mitochondrial Ca²⁺ uptake and ROS production compared with WKY controls. In an Ang II–infusion model, genetic deletion of TRPC3 attenuated mitochondrial Ca²⁺ uptake and ROS generation and ameliorated hypertension, supporting a role for mitochondrial TRPC3–dependent redox signaling in hypertensive vascular dysfunction [78] (Table 2). Parallel to TRPC3, evidence also implicates IP_3_Rs in hypertensive vascular dysfunction. Bernier and colleagues reported significantly higher IP_3_ binding capacity in aortic microsomes from SHR compared to WKY rats, indicating increased IP_3_R activity that may contribute to elevated blood pressure [23]. Moreover, IP_3_ production in aortic smooth muscle cells from SHR was greater than in WKY controls [24]. In Milan hypertensive strain (MHS) rats, augmented IP_3_R1 levels were detected in smooth muscle cell membranes from de-endothelialized mesenteric arteries [80]. Likewise, increased IP_3_R expression in mesenteric arteries from hypertensive rodent models correlated with enhanced sensitization and amplification of IP₃-dependent Ca²⁺ release [81]. Importantly, genetic loss of all three IP_3_R subtypes in VSMCs markedly reduced contractile responses to multiple vasoconstrictors and significantly attenuated the angiotensin II–induced rise in systolic blood pressure, supporting an essential role for VSMC IP_3_R–mediated Ca²⁺ release in angiotensin II–dependent hypertension [10] (Table 3). Collectively, these studies demonstrate that hypertensive models consistently exhibit upregulation of TRPC3 and/or IP_3_R expression across multiple vascular beds. Beyond hypertension, VSMC IP_3_R1 has also been shown to act as a key mediator of increased vascular tone in heart failure, promoting elevated afterload and cardiac decompensation during disease evolution [82]. The elevation of TRPC3 channels and their molecular coupling to IP_3_Rs enhances vasoconstriction induced by endothelin-1 during hypertension. Both IP_3_ and endothelin-1 stimulate this coupling, leading to activation of nonselective cation currents in mesenteric artery myocytes. Notably, TRPC3 protein levels are significantly elevated in mesenteric arteries from SHR compared to normotensive controls, and the density of IP_3_-evoked nonselective cation currents is also increased in SHR myocytes. This likely reflects a greater number of TRPC3 channels in close proximity to IP_3_R1 and enhanced molecular coupling [25].

Although evidence supports upregulated TRPC3 and/or IP_3_R signaling in hypertension, only a limited number of studies have directly examined TRPC3–IP_3_R coupling in hypertensive vessels, highlighting the need for further investigation of the contribution of TRPC3–IP_3_R coupling to vascular tone and blood pressure elevation.

Beyond animal models, human studies also support a role for TRPC3 in the pathogenesis of hypertension. Hu and colleagues reported that the increased TRPC3 mRNA expression in peripheral blood mononuclear cells (PBMCs) from hypertensive patients correlates with dietary salt intake and is significantly associated with systolic blood pressure. These findings suggest that TRPC3 may contribute to the development of salt-sensitive hypertension in humans [83]. Both human and rat experimental studies have demonstrated elevated TRPC3 expression in the context of pulmonary arterial hypertension (PAH). In pulmonary arterial smooth muscle cells from PAH models, TRPC3 contributes to dysregulated store-operated calcium entry, which is associated with key pathological features such as excessive cell proliferation, enhanced migratory capacity, resistance to apoptosis, and sustained vasoconstriction. Notably, in vivo pharmacological blockade of TRPC3 has been shown to attenuate disease progression in experimental PAH models, highlighting its potential as a therapeutic target [79].

**Table 2:** Summary of reported alterations in TRPC3 in vascular smooth muscle during hypertension

Authors	Model (animal or human)	Tissue/vascular bed	Alterations in TRPC3 in vascular smooth muscle
Liu et al. (2009) [12]	SHR	Aorta	↑ Expression and function
Chen et al. (2010) [76]	SHR	Mesenteric arterioles	↑ Expression
Noorani et al. (2011) [77]	SHR	Carotid arteries	↑ Expression and function
Adebiyi et al. (2012) [25]	SHR	Mesenteric arteries	↑ Expression
Wang et al. (2017) [78]	SHR TRPC3 knockout mice	Whole-cell and mitochondrial levels in primary cultured VSMCs.	↑ Expression (SHR) Attenuated Ang II–induced hypertension (TRPC3 knockout)
Masson et al. (2023) [79]	Human and Wistar rat pulmonary arterial hypertension	Pulmonary arteries	↑ Expression

**Table 3:** Summary of reported alterations in IP_3_/IP_3_R signaling in vascular smooth muscle during hypertension

Authors	Model (animal)	Tissue/vascular bed	Alterations in IP_3_ and/or IP_3_*R* in vascular smooth muscle
Bernier S, Guillemette G.(1993) [23]	SHR	Aorta microsomes Vascular smooth muscle	↑ IP_3_ binding capacity IP_3_R activity
Wu L, Champlain J. (1996) [24]	SHR	Aortic smooth muscle cells	↑ IP_3_ production
Adebiyi et al. (2012) [25]	SHR	Mesenteric artery myocytes	↑ Density of IP₃-evoked nonselective cation currents
Abou-Saleh et al. (2013) [81]	Hypertensive mouse and rat	Mesenteric arteries	↑ IP_3_R expression
Lin et al. (2016) [10]	Smooth muscle–specific IP3R triple-knockout mouse model	Aorta and mesenteric arteries	↓ Vascular smooth muscle contractility Angiotensin II–induced increase in systolic blood pressure

### Outstanding questions and future directions

Despite evidence supporting functional coupling between TRPC3 channels and IP_3_R, relatively few studies have directly investigated this interaction in native vascular smooth muscle. Most recent work has extensively characterized TRPC3 and IP_3_R function individually, whereas few studies have specifically examined the molecular basis and functional consequences of their interaction as an integrated signaling pathway.

An aspect that is still incompletely understood is how this coupling is regulated under physiological conditions. GPCR agonists such as endothelin-1 and angiotensin II are known to increase IP_3_ production; however, it is unclear whether these stimuli also promote structural reorganization or post-translational modifications that facilitate TRPC3–IP_3_R interaction. In addition, the contribution of mechanical factors, such as vascular stretch and intraluminal pressure, has not been extensively explored, particularly in the context of hypertension.

It remains unclear whether different IP_3_R isoforms differentially contribute to TRPC3-mediated signaling in vascular smooth muscle. Although IP_3_R1 has been the most extensively studied, the potential involvement of IP_3_R2 and IP_3_R3 in TRPC3 coupling, particularly in a tissue- or disease-specific context, remains largely unexplored.

At the molecular level, the mechanisms governing TRPC3–IP_3_R coupling remain incompletely defined. Critical aspects, including binding interfaces, coupling stoichiometry, and the temporal dynamics of complex formation, are still poorly understood. These limitations likely reflect the technical challenges associated with studying transient interactions within nanoscale microdomains.

Recent advances in structural and biochemical approaches, including the purification of TRPC3 under more native lipid conditions [84], may help overcome some of these barriers and provide new insights into the molecular basis of TRPC3–IP_3_R interaction.

Emerging evidence suggests that this coupling occurs within spatially restricted Ca²⁺ microdomains, where local signals may regulate both TRPC3 activity and IP_3_R function. However, it remains unclear whether TRPC3–IP_3_R coupling operates as a compartmentalized pathway without elevating global cytosolic Ca²⁺ or instead propagates beyond these local microdomains. Limited studies suggest a role for TRPC3–IP_3_R coupling in vascular dysfunction in hypertension; further investigation is needed to more precisely define its contribution.

Future studies combining high-resolution imaging, functional assays, and targeted molecular approaches will be essential to characterize the spatial organization and regulatory mechanisms of TRPC3–IP_3_R signaling, as well as to better understand the molecular interactions and signaling pathways in vascular smooth muscle.

## Conclusion

Collectively, current evidence supports a model in which TRPC3–IP_3_R coupling functions as a localized signaling module that integrates intracellular Ca²⁺ release with membrane depolarization to amplify vasoconstrictor responses. Rather than operating as independent pathways, these proteins act within specialized ER–PM microdomains to generate spatially localized Ca²⁺ signals. This integrated framework provides a unifying perspective on Ca²⁺ signaling in vascular smooth muscle and suggests that TRPC3–IP_3_R coupling may represent a promising therapeutic target in hypertension, although the current evidence remains limited. Notably, targeting this coupling mechanism may offer greater specificity than conventional strategies that broadly inhibit Ca²⁺ influx pathways. Despite this growing body of evidence, however, TRPC3–IP_3_R coupling remains insufficiently characterized in native vascular tissues, particularly under hypertensive conditions, highlighting the need for further investigation.

## Data Availability

No datasets were generated or analysed during the current study.
